# Neural synchrony in cortical networks: mechanisms and implications for neural information processing and coding

**DOI:** 10.3389/fnint.2022.900715

**Published:** 2022-10-03

**Authors:** Kai S. Gansel

**Affiliations:** Independent Researcher, Usingen, Germany

**Keywords:** synchrony, cell assembly, oscillation, STDP, spike pattern, temporal compression, neocortex

## Abstract

Synchronization of neuronal discharges on the millisecond scale has long been recognized as a prevalent and functionally important attribute of neural activity. In this article, I review classical concepts and corresponding evidence of the mechanisms that govern the synchronization of distributed discharges in cortical networks and relate those mechanisms to their possible roles in coding and cognitive functions. To accommodate the need for a selective, *directed* synchronization of cells, I propose that synchronous firing of distributed neurons is a natural consequence of spike-timing-dependent plasticity (STDP) that associates cells repetitively receiving temporally coherent input: the “synchrony through synaptic plasticity” hypothesis. Neurons that are excited by a repeated sequence of synaptic inputs may learn to selectively respond to the onset of this sequence through synaptic plasticity. Multiple neurons receiving coherent input could thus actively synchronize their firing by learning to selectively respond at corresponding temporal positions. The hypothesis makes several predictions: first, the position of the cells in the network, as well as the source of their input signals, would be irrelevant as long as their input signals arrive simultaneously; second, repeating discharge patterns should get compressed until all or some part of the signals are synchronized; and third, this compression should be accompanied by a sparsening of signals. In this way, selective groups of cells could emerge that would respond to some recurring event with synchronous firing. Such a learned response pattern could further be modulated by synchronous network oscillations that provide a dynamic, flexible context for the synaptic integration of distributed signals. I conclude by suggesting experimental approaches to further test this new hypothesis.

## Introduction

Taken on its own, i.e., without any temporal reference, a single action potential represents a single bit of information. If all brain cells were active independently, neural coding could be fully described by defining the mechanisms of spike generation and characterizing the firing patterns of single cells, and no additional information would be contained in multineuronal activation patterns. Yet, as countless studies have shown, neurons dynamically coordinate their firing. Neural coding thus involves the formation of multineuronal firing patterns that may function as discrete, information carrying elements. The organization of these elements as well as their correlation with cognitive processes constitutes the core problem in neural coding, with many details still being unclear even after decades of thorough investigation.

Delage was probably the first who anticipated ensembles of coactive neurons to be the physiological equivalent of what he called “a single idea” (Delage, 1919; Herculano-Houzel, 1999). Driven by direct interactions, the members of the ensemble would leave on the physical connections among them a trace, a “relic,” that would facilitate their future cooperation. Some 30 years later, Hebb elaborately formulated what became known as the “cell assembly hypothesis” (Hebb, 1949). He conjectured that through “some growth process or metabolic change,” repeated coactivation of a group of neurons causes the formation of a “cell assembly”—an anatomically dispersed set of neurons among which excitatory connections have been potentiated. As a consequence, repeating activation patterns in a way translate into assembly formation, and are henceforth represented by the activity of the assembly. Given that repeating excitation patterns most likely carry some meaning, each cell assembly is proposed to be a correlate of some discrete, cognitively meaningful item of information. Hebb’s concept has been reviewed many times and refined ever since (Braitenberg, 1978; Sakurai, 1999; Harris, 2005). In particular, the strict connectivity-based definition has been relaxed in favor of a purely temporal one (Gerstein et al., 1989; Fujii et al., 1996; Singer et al., 1997). From a downstream point of view, there is no need for the neurons in the assembly to be directly connected—all that matters is their synchronous activity within a critical time window.

Taking for granted that irreducible cognitive contents cannot be represented by the activity of single cells, but are based on the transient coactivation of confined groups of neurons (Singer, 2007), we are thus left with the question of how long this critical time window would be, and how precisely the firing of the constituent neurons would be coordinated. Considering that the temporal scale of neuronal synchronization would have to comply with even the fastest cognitive processes, the answer comes down to at most a few milliseconds, or at least, the shorter the better. In this respect, it just fits that synchronization of neuronal discharges on the milliseconds scale has long been found to be a prevalent and functionally important attribute of neural activity (Singer, 1993; Stevens and Zador, 1998; Usrey and Reid, 1999; Lestienne, 2001; DeWeese and Zador, 2006; Uhlhaas et al., 2009; Gansel, 2014).

The aim of this article is to review classical concepts and corresponding evidence of the mechanisms that govern the synchronization of distributed discharges in cortical networks and to relate those mechanisms to their possible roles in coding and cognitive functions. To this end, the prerequisites for precise coordination of multineuronal firing on the molecular, cellular, and network level, with a special emphasis on network oscillations, are discussed (sections “Prerequisites of a directed synchronization of distributed spiking activity” and “The role of neural oscillations”). To accommodate the need for a selective, *directed* synchronization of cells and to explain the discovery of recurring patterns of precisely synchronous, distributed spiking activity in the neocortex in the absence of network oscillations and sensory input (Gansel, 2014), the new “synchrony through synaptic plasticity” hypothesis is presented (section ‘The “synchrony through synaptic plasticity” hypothesis’). The hypothesis is inspired by theoretical studies that have demonstrated that neurons equipped with spike-timing-dependent plasticity (Caporale and Dan, 2008) may tune to repeating spatiotemporal input patterns by potentiating synaptic weights on afferents that consistently fire early, thereby steadily decreasing postsynaptic response latency with respect to the onset of the pattern, until it reaches a minimal value (Guyonneau et al., 2005; Masquelier et al., 2008, 2009). Given appropriate input firing patterns and plasticity mechanisms, multineuronal spike sequences should therefore progressively be compressed in time and eventually become synchronized (Suri and Sejnowski, 2002), if the participating neurons respond to coherent input. Finally, the integration of synchronizing mechanisms is discussed with respect to their possible roles in cognitive functions, referring to Chalmers’ “principle of structural coherence” (Chalmers, 1995; section “Neural correlates of cognitive processes”). I conclude by contemplating the consequences of the formation of synchronous cell assemblies on neuronal coding and by suggesting experimental approaches to further test the new “synchrony through synaptic plasticity” hypothesis (section “Discussion”).

## Prerequisites of A Directed Synchronization of Distributed Spiking Activity

### Timescales and accuracy of neuronal signaling

Energy supply critically limits signaling in the brain (Karbowski, 2014). For the cerebral cortex, the volume of signal traffic that can be supported by the brain’s metabolic rate was calculated to be about five action potentials per neuron and second in rat and less than one per neuron and second in human (Laughlin and Sejnowski, 2003; Lennie, 2003). Considering the speed of neural computations, the permissible signaling rate is remarkably low, and this metabolic limit must affect the way in which information is processed. Recordings from sensory cortices suggest that the nervous system has countered this natural constraint by distributing signals sparsely in time and space (Weliky et al., 2003; Kerr et al., 2005; DeWeese and Zador, 2006; Waters and Helmchen, 2006; Hromádka et al., 2008; Jadhav et al., 2009). The conclusion that at most a few discharges per neuron are available to convey a message is confirmed by the finding that sensory information is transmitted quickly along feed-forward connections (Thorpe et al., 1996), requiring only 10–15 ms per processing stage (Tovée, 1994). Therefore, it was argued that information can only be represented by short, fast responses forming a sparse population code. In fact, reliable decoding of stimulus features is possible based on the relative timing of the first spikes elicited in individual neurons in the retina (VanRullen and Thorpe, 2001; Gollisch and Meister, 2008), the olfactory system (Junek et al., 2010), the somatosensory system (Petersen et al., 2001; Johansson and Birznieks, 2004), and even in cell cultures (Shahaf et al., 2008). But how reliable is the initiation of action potentials in single neurons, and what is their temporal precision? Membrane potential fluctuations induced by stochastic ion channel gating and probabilistic release of synaptic vesicles are potential sources of random variations in spike generation and timing (Faisal et al., 2008; Ribrault et al., 2011). So, the probability that an arriving presynaptic nerve impulse fails to evoke a postsynaptic response is remarkably high, between 0.5 and 0.9 (Allen and Stevens, 1994; Laughlin and Sejnowski, 2003). However, because of the great number of synapses, failures do not necessarily lose information. The variability introduced by nondeterministic processes acting on the level of single molecules may average out on the cellular level (Atmanspacher and Rotter, 2008) and may even sharpen the signal due to stochastic resonance (McDonnell and Abbott, 2009; McDonnell and Ward, 2011). The amplitude and exact timing of somatic potentials in response to a particular input would be expected to approach a Gaussian distribution, giving rise to precisely timed action potentials in most cases while occasionally failing to cause a spike in time. This is indeed what could be observed by repeatedly injecting irregular depolarizing currents into cortical neurons *in vitro* (Mainen and Sejnowski, 1995), and simulations suggest that the same is true for the axonal propagation of action potentials, leading to small, mostly submillisecond variations in spike timing over distances of millimeters (Faisal and Laughlin, 2007). High reliability of spiking has also been demonstrated in the visual (Kara et al., 2000; Herikstad et al., 2011) and in the auditory system (DeWeese et al., 2003) *in vivo*.

### Cellular properties impacting precision of spiking

The temporal precision of neuronal communication crucially depends on a number of basic cellular properties. Spike-timing-dependent plasticity rules for modifications in synaptic strength indicate that postsynaptic potentials are effectively integrated within only 20–30 ms (Dan and Poo, 2006; Caporale and Dan, 2008). Such short integration times mainly result from rapidly deactivating AMPA receptors that can have deactivation time constants of less than a millisecond (Hestrin, 1993; Geiger et al., 1997; Magee, 2000) and indirectly control the kinetics of NMDA receptor currents by only allowing for a correspondingly short release of the magnesium block (Oertner, 2009; Holbro et al., 2010). In addition, disynaptic feedforward inhibition may confine the effective integration time window in the soma to a few millisecond (Pouille and Scanziani, 2001). Backpropagating action potentials coinciding at the synapse with excitatory postsynaptic potentials may trigger dendritic calcium spikes and in this way cause highly nonlinear responses (Stuart et al., 1997; Schiller et al., 1998; Larkum et al., 1999; Stuart and Häusser, 2001; London and Häusser, 2005). Another nonlinear element is the spike threshold which is inversely related to the rate of membrane depolarization preceding a spike and the rise time of the action potential, endowing neurons with enhanced sensitivity to synchronous inputs (Azouz and Gray, 2000; Harnett et al., 2012; Fontaine et al., 2014). With increasing input rates, both the amplitude and duration of somatic potentials in response to synaptic input are reduced, resulting in a shortening of the temporal integration window and requiring a yet higher precision of presynaptic signals to drive the neuron to fire (Azouz and Gray, 2003; Léger et al., 2005). The dynamics of spike threshold adaptations most likely depend on fast sodium channel inactivation following membrane potential fluctuations (Platkiewicz and Brette, 2011), making neurons particularly susceptible to rapid input fluctuations (Mensi et al., 2016) while slow voltage fluctuations do not contribute to spiking because they are filtered by threshold adaptation. Finally, many synapses operate most reliably at certain frequencies of presynaptic firing and display depression or facilitation of postsynaptic responses (Galarreta and Hestrin, 1998; Reyes et al., 1998; Thomson, 2003). Such synapses effectively detect changes in the firing rate, but report frequency of maintained activity poorly because they are unable to respond at sustained rates (Abbott et al., 1997; Tsodyks and Markram, 1997; Avissar et al., 2007). Besides the effects of repetitive signaling on the release probability of vesicles at the presynapse, the kinetics of transmitter binding and channel gating of postsynaptic NMDA receptors produces currents with distinct waveforms depending on pulse frequency (Popescu et al., 2004), leading to the long-known fact that the postsynaptic response is sensitive to the exact timing of successive input signals (Reich et al., 2000). One important consequence of this sensitivity is that modifications in synaptic strength depend not only on the relative spike timing between the neurons but also on the spiking pattern within each neuron, with the timing of the first spike in each burst being dominant in determining the ensuing synaptic modifications (Froemke and Dan, 2002). Taken together, these properties make neurons susceptible to transient signals and precise spike timing codes, and the transmission of a continuous rate signal difficult (König et al., 1996).

### Counting spikes vs. spikes that count

Nevertheless, dissenting models have been devised in which the membrane potential undergoes a random walk to the spike threshold so that any temporal structure in the input is lost (Shadlen and Newsome, 1998). Based on these models, it has been concluded that the summation of postsynaptic potentials in cortical neurons is too imprecise to support precise spike timing codes, thus leaving as the only coding dimension the firing rates of neurons. A reliable estimate of the instantaneous firing rate would then require the simultaneous readout of a population of neurons (Gautrais and Thorpe, 1998), implying ergodicity and independence of cells. Assuming uncorrelated inputs, the model by Shadlen and Newsome predicts a Gaussian distribution of membrane potential with only small membrane potential fluctuations. *In vivo* recordings, however, revealed highly non-Gaussian membrane potential dynamics displaying quiescent periods interrupted by large, brief excursions consistent with coordinated presynaptic firing (DeWeese and Zador, 2006). As has been shown both theoretically and experimentally, the organization of presynaptic input into synchronous volleys is also necessary to explain the irregular output firing of neurons (Softky and Koch, 1993; Stevens and Zador, 1998). These findings are in conflict with basic assumptions of the model and question its validity. Moreover, correlations between cells would compromise the ensemble representation of firing rate especially at high frequencies, imposing rigorous constraints on the temporal accuracy of neural computations (Mazurek and Shadlen, 2002). The above arguments indicate that neuronal dynamics and theoretical considerations may be at odds with a firing rate code. Further there is evidence for millisecond-precise spike timing depending on sensory input, behavior, or internal state in a variety of cortical areas like the frontal cortex (Vaadia et al., 1995), the motor cortex (Riehle et al., 2000; Hatsopoulos et al., 2003; Shmiel et al., 2005, 2006), the somatosensory cortex (Foffani et al., 2004; Panzeri and Diamond, 2010), auditory cortex (deCharms and Merzenich, 1996; Engineer et al., 2008), visual cortex (Gray et al., 1992; König et al., 1995; Bair and Koch, 1996; Tiesinga et al., 2008), and also in upstream and downstream areas like the thalamus (Dan et al., 1998; Usrey, 2002; Desbordes et al., 2008), retina (Berry et al., 1997; Greschner et al., 2006; Jacobs et al., 2009), and the hippocampus (Harris et al., 2003; Robbe et al., 2006). We are thus led to a view of neural activity as being basically and essentially characterized by sparse, temporally precise, coordinated firing.

### Coordinating neuronal activity: mechanisms and functions

Sparse activation of small neuronal populations and even of single cells in the neocortex has been shown to evoke distinct movements (Brecht et al., 2004) and actually drive behavior (Houweling and Brecht, 2008; Huber et al., 2008), demonstrating a possible functional role of sparse cortical activity that might be explained by the ability of single discharges to initiate both widespread excitation and inhibition (Kapfer et al., 2007; Molnár et al., 2008; Li et al., 2009; Wolfe et al., 2010). It has been argued that this sensitivity of the cortical network to single action potentials would cause relatively large random membrane potential fluctuations and so entail a reduction of the signal-to-noise ratio in neuronal communication (London et al., 2010), implicitly assuming that spike generation is inherently noisy. Reversing the argument, however, one might as well conclude that this very sensitivity requires signal propagation to be accurately controlled, and one might suspect that the brain has evolved to make optimal use of its limited resources and has developed adaptive mechanisms to prevent the processing of signals that carry no meaning or, even worse, affect information processing. Cortical computation would then imply sparse representations and a very selective routing of signals. To integrate and segregate distributed information efficiently, neurons would have to coordinate their firing and engage in coherent activity patterns while maximizing the repertoire of functional states (Tononi et al., 1998; Sporns and Kötter, 2004), which amounts to operating in a critical regime between total independence and perfect functional unity. Not surprisingly, the brain is endowed with a variety of features and components controlling neuronal cooperation and the propagation of neuronal activity.

### Elements controlling neuronal signaling and cooperation

The diversity of neuronal cell types and the intrinsic heterogeneity of their biophysical properties have been found to increase the system’s coding capacity through a decorrelation of the firing of cells (Padmanabhan and Urban, 2010). Likewise, the dynamics in neuronal populations become more complex through the divergent and convergent actions of various transmitters and neuromodulators (Marder and Thirumalai, 2002; Edeline, 2012). Another factor that has an impact on the functional repertoire of the network is the mere number of its neurons and the degree of their connectedness. These and other cellular and network properties also play a role in regulating the activity flow in the brain. First and foremost, the propagation of signals is confined by the functional anatomy of the network, that is, synaptic connections and their effective strengths (Sporns et al., 2000). Given the dense meshwork of axonal and dendritic processes, neural signals are often presumed to potentially take any direction at any time; however, they do not. Rather, synapses may temporarily be silenced (Voronin et al., 1996), and the overall distribution of synaptic strengths in local cortical circuits comprises relatively few strong connections embedded in a “sea” of weaker ones (Song et al., 2005; Yassin et al., 2010), constraining the range of possible signaling pathways (Luczak and MacLean, 2012) and again maximizing the network’s capacity to produce and retain stable activity patterns (Chen et al., 2010). Besides the effective synaptic connectivity, a neuron’s functional state is relevant to the routing of signals. In particular, neuronal responsiveness is controlled through the rate and balance of excitatory and inhibitory inputs (Haider and McCormick, 2009). Synaptic bombardment can cause continuing depolarizations and an increased variance of the membrane potential and thereby raise a neuron’s sensitivity especially to inputs of small amplitude (McCormick et al., 2003; Shu et al., 2003), while the concomitant drop in input resistance leads to substantial dendritic attenuation of electrical signals, with distal synapses having reduced effects at the soma (Destexhe and Paré, 1999; Fellous et al., 2003). This high conductance state (Destexhe et al., 2003) is thought to be generated through local recurrent excitation (Sanchez-Vives and McCormick, 2000) and has been linked to the encoding of sensory information in the primary visual cortex (Anderson et al., 2000). In addition to these input-driven fluctuations between a hyperpolarized “down” state and a depolarized “up” state, the intrinsic ability of neurons to respond selectively to inputs at preferred frequencies (Hutcheon and Yarom, 2000) affects their integrative properties: Both synaptic mechanisms (Thomson, 2003) and ionic conductances (Llinás, 1988) may create a resonance effect (Hutcheon et al., 1996; Ulrich, 2002) that influences spike timing and information transmission between cells (Fellous et al., 2001). Finally, the propagation of signals directly depends on the ongoing activity pattern, with neuronal oscillations playing a prominent role in defining temporal windows for effective excitation (Fries, 2005, 2009; Fries et al., 2007; Cardin et al., 2009). The interplay of concurrent excitatory and inhibitory inputs (Isaacson and Scanziani, 2011), in conjunction with ephaptic transmission of electrical potentials (Weiss and Faber, 2010; Anastassiou et al., 2011), dynamically determines the possible impact of incoming signals (Chance et al., 2002; Azouz and Gray, 2003). Acting together, all these structural and functional elements gate the information flow in the brain (Jefferys, 1995; Salinas and Sejnowski, 2001; Vogels and Abbott, 2009; Adesnik and Scanziani, 2010; Fröhlich and McCormick, 2010) and produce both the network and the activity patterns that then give rise to cognitive functions. In the following sections, I will further discuss the role of synchronous oscillatory activity therein.

## The Role of Neural Oscillations

### Theoretical considerations

Synchronously discharging neurons often produce oscillatory rhythms of various frequencies, generated by networks of diverse sizes (Kopell et al., 2000; Buzsáki and Draguhn, 2004; Buzsáki, 2006). Theoretically, synchronous oscillations might simply be an unavoidable byproduct of neuronal network dynamics without any particular computational role. Alternatively, they could directly contribute to the representation of information, for example by providing the timing for an internal clock or as a reference signal relative to which spike times become meaningful, or they could actively regulate the flow of information in neural circuits by interfering with the action potential generation and temporally link neurons into assemblies (Buzsáki and Draguhn, 2004; Sejnowski and Paulsen, 2006; Fries et al., 2007). Encoding by phase and synchrony has highly attractive computational properties (Hopfield, 1995, 2004; Nádasdy, 2009). It has been proposed that phase encoding might affect the temporal segmentation of several working memory items (Lisman and Idiart, 1995; Jensen, 2006), and that waves of activity might serve to tag sensory input at different spatial locations with a unique phase (Ermentrout and Kleinfeld, 2001). The addition of phase information may be used as a means to segment and categorize parallel inputs. In a similar way, top-down processes could shape spiking activity by coordinating subthreshold membrane potential fluctuations to establish selective functional relationships between neurons during states of anticipation (Engel et al., 2001). The idea that the formation of dynamic links mediated by synchrony over multiple frequency bands subserves neuronal communication (Bressler et al., 1993; Singer, 1999b; Varela et al., 2001; Averbeck and Lee, 2004) was dubbed “communication through coherence” by Fries (Fries, 2005). Rhythmic excitability fluctuations are thought to confine neural signal transmission such that only coherently oscillating neuronal groups can interact effectively, in the sense that their excitability peaks need to coincide to facilitate the propagation of spikes. The resulting effective communication structure may flexibly be rearranged through shifts in attention or other cognitive processes that come along with alterations in the oscillation patterns, which in turn would alter the selective linking of distributed representations (Serences and Yantis, 2006).

### Binding by synchrony

Coherent oscillations could also provide a mechanism to solve the so-called “binding problem” (Treisman, 1996): If we assume that some irreducible percept or thought or motor plan is represented by a group of neurons on a dynamical basis, what is the signature that transiently binds their activity into a unified whole? Milner proposed that cells selectively segregate their firing in time to signal their functional relationships (Milner, 1974), and von der Malsburg formulated the “correlation theory of brain function” based on the same rationale (von der Malsburg, 1986; von der Malsburg and Schneider, 1986; von der Malsburg, 1995). Singer and co-workers adopted these concepts (Singer and Gray, 1995; Engel et al., 1997; Gray, 1999) and advanced the “binding by synchrony” hypothesis that suggests that functional relations between neurons are encoded by synchronous firing in the millisecond range, brought about by the phase-locking of distributed oscillations (Engel et al., 1992; Singer, 1993, 1999a). The idea behind this is that elementary relations are represented by the firing of individual neurons mediated through appropriate convergence of input connections, and that more complex relations are represented by the activity of cell assemblies generated by dynamic associations of cells (Singer et al., 1997; Roelfsema, 2006).

### Correlating phase of firing with cognitive performance

Network oscillations are often carried by rhythmic inhibitory input originating from synchronized interneuronal spiking (Buzsáki and Chrobak, 1995; Whittington et al., 2000; Fricker and Miles, 2001; Hasenstaub et al., 2005; Bartos et al., 2007; Sohal et al., 2009). It has been shown both experimentally (Lampl and Yarom, 1993; Volgushev et al., 1998; Schaefer et al., 2006; McLelland and Paulsen, 2009) and in simulations (Hopfield, 2004) that the ensuing subthreshold membrane potential fluctuations interact with excitatory inputs such that the timing of action potentials becomes a function of the oscillation phase and is made less variable. As a result, discharges are temporally coordinated and related to the network rhythm. Examples in which the phase of firing carries significant information have been reported from prefrontal (Siegel et al., 2009), auditory (Kayser et al., 2009), and visual cortex (Montemurro et al., 2008). In cortical area V4 of monkeys, the frequency-dependent strength of the phase-locking of spikes is modulated by attention (Fries et al., 2001; Grothe et al., 2012). Moreover, the interaction of neuronal groups has been found to depend on the phase relation between rhythmic activities within the groups (Womelsdorf et al., 2007), consistent with the idea that firing phases and times of increased susceptibility to input need to match the inter-group transmission delays to facilitate the propagation of spikes. Similarly, the strength of the interareal phase synchronization of neuronal activity in monkey V4 and prefrontal cortex was shown to correlate with visual short-term memory performance (Liebe et al., 2012), suggesting that this synchronization subserves intercortical communication and contributes to the maintenance of visual short-term memories. Thus, neural oscillations may dynamically shape suprathreshold activity and flexibly arrange signaling pathways in concert with cognitive processes (Wang, 2010; Cannon et al., 2014).

### Attempts at a causal proof

Given that the brain, like every system which has opposing forces such as excitation and inhibition, almost inevitably will generate oscillations (Buzsáki, 2006; Wang, 2010), it is hard to believe that it did not evolve to make use of them. But how exactly do neural oscillations relate to the processing of information? The idea that oscillations could serve as an internal clock has been dismissed in favor of a model using high-dimensional network states for encoding time (Mauk and Buonomano, 2004; Karmarkar and Buonomano, 2007). The difficulty in assigning functional relevance to synchronous oscillations lies in the correlative nature of most of the investigations done so far. There are some exceptions, though. In a series of experiments, Laurent and colleagues used picrotoxin (a GABA antagonist) to disrupt synchronous oscillations in the olfactory systems of insects (MacLeod and Laurent, 1996) and so were able to demonstrate that the selective desynchronization of projection neurons in the antennal lobe degrades the selectivity of downstream neurons (MacLeod et al., 1998) and impairs the animal’s ability to discriminate molecularly similar odorants (Stopfer et al., 1997). In mammals, however, the situation is less clear. Mice lacking the GABAA receptor β3 subunit produce enhanced oscillations in the olfactory bulb and after training are better than normal in discriminating closely related odorants but worse in discriminating odorant mixtures (Nusser et al., 2001). In the rat olfactory bulb, oscillatory power appears to be actively modulated depending on the molecular similarity of odorants that the rat has to distinguish, suggesting a role of enhanced network oscillations in stimulus disambiguation (Beshel et al., 2007). On the other hand, newborn rats who have very few GABAergic granule cells do not produce synchronous oscillations and yet are as good at making odor discriminations as older ones who have developed interneurons and do produce oscillatory activity in response to a stimulus (Fletcher et al., 2005). The most convincing studies so far that tried to establish a causal link between synchronous oscillations and behavioral performance in vertebrates again relied on pharmacological or optogenetic interference with normal neuronal functioning: In the frog retina, a subclass of oscillating ganglion cells responding to expanding dark objects gets out of sync when exposed to bicuculline (GABAA antagonist), leading to the failure of an escape behavior as it is normally induced by such stimuli (Ishikane et al., 2005). In the rat hippocampus, cannabinoids cause a decrease in oscillatory power in various frequency bands without affecting average firing rates, which in turn impairs memory formation (Robbe et al., 2006). Conversely, memory consolidation can be rescued through optogenetic entrainment of hippocampal network oscillations in sleep-deprived and amyloid precursor protein overexpressing mice (Ognjanovski et al., 2018; Etter et al., 2019). These examples show that neural network function is often associated with neuronal oscillations, but in most cases, it is unclear how exactly they contribute to the processing of information. Nevertheless, on a mechanistic level, they play a consequential role in coordinating multineuronal activity.

### Cellular mechanisms: a deeper look

Network oscillations naturally arise from the interplay of recurrent excitatory and inhibitory connections and the resonant properties of individual neurons (Llinás, 1988; Gray and McCormick, 1996; Hutcheon and Yarom, 2000; Cardin et al., 2005). Since they are a built-in feature of virtually every neural system, it can be supposed that controlling them is one of the brain’s most basic functions (Buzsáki, 2006). Cortical networks generate synchronous discharges at various frequencies involving variable numbers of cells and thereby distinguish varying functional states for the processing of information (Buzsáki and Draguhn, 2004). By rapidly balancing excitation with inhibition, the oscillation frequency can be instantaneously modulated (Atallah and Scanziani, 2009). In participating cells, rhythmic synaptic inputs and oscillations of the local electrical field restrict effective excitation to the depolarizing phase of the oscillation cycle, thus adding a dynamic, temporal gate for the transmission of signals to the spatial gates given by the functional connections in the network (Fries, 2009). Depending on the interactions of concurrent rhythms (Lakatos et al., 2005; Canolty et al., 2006; Roopun et al., 2008), spiking activity may hence be orchestrated on multiple spatiotemporal scales in parallel by modulations of the phase and amplitude of distributed oscillations (Jensen and Colgin, 2007; van Aerde et al., 2009; Canolty et al., 2010; Kopell et al., 2010), possibly even across subjects when they engage in coordinated actions (Lindenberger et al., 2009).

### Temporal coding vs. binary coding

Taking a closer look at how oscillatory network activity interferes with a single cell’s firing, the question arises to what extent the timing of an action potential can be controlled by oscillatory input. It is known that through the interplay between the magnitude of dendritic excitation and rhythmic inhibition of the somatic region, the more excited cells tend to fire earlier in the oscillation cycle (Volgushev et al., 1998; Vinck et al., 2010), such that the phase of firing corresponds to the excitatory drive of the neuron (McLelland and Paulsen, 2009). On these grounds, it has been proposed that the interaction of subthreshold membrane potential oscillations with incoming excitatory signals could serve as a fundamental computational mechanism for the implementation of a temporal coding scheme in which information is encoded by the precise timing of a spike relative to the phase of the ongoing oscillation (Hopfield, 1995; Fries et al., 2007; Nádasdy, 2009). Yet, although the particular phase in which a neuron fires can contain significant information (Montemurro et al., 2008; Kayser et al., 2009; Siegel et al., 2009), such a coding scheme would necessarily be limited to the timescale on which rhythmic membrane potential fluctuations can advance or delay the spike timing in a systematic way without completely suppressing spike generation. *In vitro* recordings have shown that this timescale dynamically depends on the average absolute membrane potential, the time constant of the membrane, the strength of the input signal, and the frequency and amplitude of the membrane potential fluctuations (Volgushev et al., 1998; McLelland and Paulsen, 2009). According to these studies, only neurons receiving tonic excitatory drive, combined with slow oscillatory input having a relatively long period compared to the membrane time constant, may produce output signals whose timing is smoothly scaled across the whole depolarizing phase. If, however, there is only transient excitatory input or the period of the rhythmic modulation approaches the time constant of the membrane, neural oscillations act essentially as a logic gate relaying incoming excitatory signals only within accordingly narrow time windows. In so doing, network oscillations provide context to afferent signals by selectively routing information in the brain in a dynamic and state-dependent way. Playing a complementary role to neuronal connectivity, rhythmic modulations of the membrane potential may also synchronize multineuronal firing when paired with prolonged excitatory input; in this case, spike timing is largely determined and actively controlled by the phase of the modulation and the overall activation level of the cell (Lampl and Yarom, 1993; Volgushev et al., 1998; Markowitz et al., 2008). In addition, oscillations of the membrane potential may improve action potential precision by imposing defined temporal windows for the effective integration of excitatory inputs (Hopfield, 2004; Schaefer et al., 2006; Poo and Isaacson, 2009). Thus, rhythmic excitability fluctuations are able to dynamically control the routing and the timing of neuronal signals (Jacobs et al., 2007), and it might not be a coincidence that the phase of ongoing network oscillations in the human brain has been found to correlate with the perception of sensory stimuli (Luo and Poeppel, 2007; Busch et al., 2009). Whether the interference of oscillatory activity with afferent inputs and the resulting spike timing constitute a temporal code or a binary code then depends on the timescale of their effective interaction.

## The “Synchrony Through Synaptic Plasticity” Hypothesis

If the dynamic, directed synchronization of discharges in cortical networks represents the neural correlate of some forms of cognitive processing, it is evident that this synchronization cannot rely on random connectivity and on a random selection of synchronously active cells, but would have to be based on adaptive learning of repeating activity patterns to provide meaning. On these grounds, I propose the “synchrony through synaptic plasticity” hypothesis.

**Figure 1:**
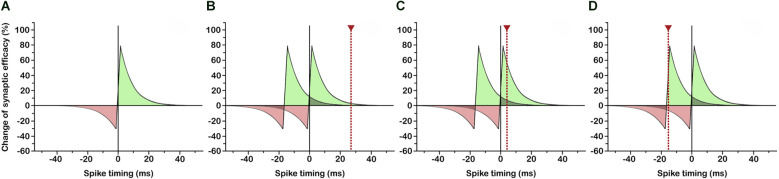
Learning to respond to the onset of a recurring input pattern through spike-timing-dependent plasticity (STDP). **(A)** Classical STDP rule: If the presynaptic input signal slightly precedes postsynaptic spiking, synaptic efficacy is enhanced (green area), if postsynaptic spiking occurs slightly before presynaptic input, synaptic efficacy is reduced (red area). Spike timing in ms indicates postsynaptic vs. presynaptic spiking. **(B)** Initial situation: Two succeeding input signals, possibly arriving at different synapses, are followed by a postsynaptic spike (dotted red line) inside the temporal window for synaptic potentiation. **(C)** Through an increase in synaptic efficacy, postsynaptic response latency is reduced, so that postsynaptic spiking falls into the temporal window for synaptic potentiation subsequent to the first input signal. **(D)** Continuing latency reduction of postsynaptic spiking leads to a response to the first input signal (=onset of recurring input pattern) with shortest possible latency.

**Figure 2:**
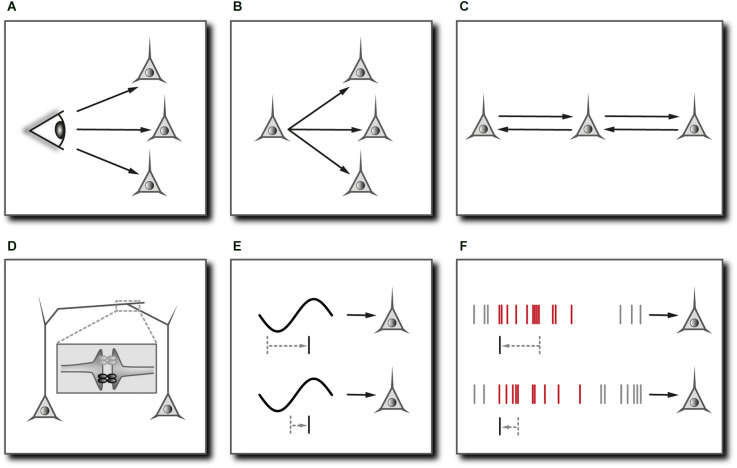
Mechanisms mediating neuronal synchronization in cortical networks. **(A)** Common sensory input. In principle, neurons may fire synchronously simply because they are driven by a common sensory stimulus. In cortical areas, however, sensory signals are modulated by the ongoing activity and follow a multitude of diverging and converging pathways with differing delays, rendering neuronal synchrony non-trivial. Prerequisite: Adaptations of the functional network to bring about synchronous groups of neurons depending on stimulus features. May require **(F)**. **(B)** Common input from the same presynaptic neuron. Prerequisite: Isochronous synaptic connections. **(C)** Dynamical relaying. Two cells/populations of cells can become synchronized with zero phase lag if coupled reciprocally to a third cell/population of cells. Prerequisite: Relay cell/population of cells occupies a temporally equidistant location from the neurons to be synchronized. Comes down to **(B)**. **(D)** Electrical coupling through gap junctions. Reciprocal transmission of graded potentials facilitates the synchronization of coupled cells especially through the formation of multi-cell syncytia, even if the impact of one cell onto another is relatively weak. Reproduced in part from Stephan et al. (2021). **(E)** Network oscillations. Subthreshold membrane potential oscillations restrict effective excitation to the depolarizing phase of the oscillation cycle and may synchronize coherently oscillating cells by actively controlling the phase of their spiking. Prerequisite: Slow oscillatory input with a relatively long period compared to the membrane time constant and tonic excitatory drive. **(F)** Synchrony through synaptic plasticity. Neurons that are excited by a repeated sequence of synaptic inputs (marked in red) may learn to selectively respond to the onset of this sequence through synaptic plasticity. Multiple neurons receiving coherent (not necessarily identical) input could thus actively synchronize their firing by learning to selectively respond at corresponding temporal positions. Prerequisite: Effective spike-timing-dependent synaptic plasticity.

In various neural circuits and a variety of species ranging from insects to humans the strengthening and weakening of synapses has been shown to depend on the relative timing of pre- and postsynaptic spiking in narrow time windows (Figure 1A; Bi and Poo, 2001; Dan and Poo, 2006; Mansvelder et al., 2019) that in turn depend on dendritic location (Froemke et al., 2005, 2010; Letzkus et al., 2006; Sjöström and Häusser, 2006) and the amplitude and decay time constant of the postsynaptic potential (Fuenzalida et al., 2007). This so-called spike-timing-dependent plasticity (Caporale and Dan, 2008) has profound implications for the shaping of the functional neuronal network and the synchronization of distributed discharges. Under conditions in which synaptic potentiation occurs if incoming signals slightly precede postsynaptic depolarization (Figure 1B), inputs that consistently fire the postsynaptic neuron with short latency develop strong synapses, while synapses of less effective inputs are weakened (Markram et al., 1997; Song et al., 2000). As a consequence, response latencies of potentiated synapses become shortened (Boudkkazi et al., 2007), causing a backward shift of the critical time window and bringing earlier inputs into effect (Figure 1C). In this way, neurons could become responsive to ever earlier signals of a recurring input pattern, so long as the temporal delay between succeeding input spikes does not exceed the critical time window for synaptic plasticity (Figure 1D). Multiple neurons being consistently driven by (parts of) the same repeating spike pattern could thus actively synchronize their firing by learning to selectively respond to this pattern at corresponding temporal positions (Figure 2F). This possibly gets to the core of what assembles cell assemblies and presents a simple and effective mechanism for coordinating multineuronal activity in the brain.

### Relation to existing work

**Figure 3:**
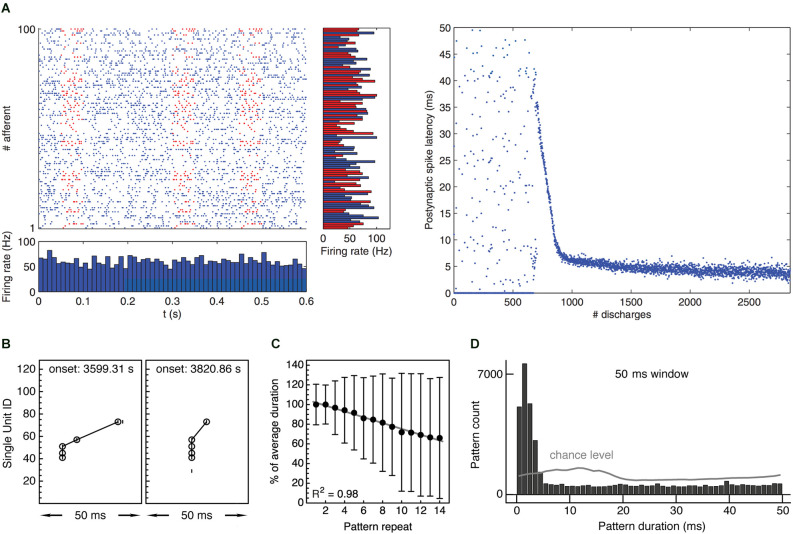
Experimental evidence supporting the “synchrony through synaptic plasticity” hypothesis. **(A)** Left: Simulation showing 100 afferent inputs to a model neuron equipped with STDP. Half of the input spike trains contain a repeating spatiotemporal activity pattern lasting about 50 ms. Right: After an initial unselective period, the receiving neuron learns to selectively respond to the repeating pattern, gradually reducing response latency with respect to pattern onset from about 35 ms to about 5 ms, thus “tracking back” through the pattern and becoming a fast and reliable pattern detector. Taken from Masquelier et al. (2008). **(B)** Two occurrences of an example spike pattern recorded from spontaneous multineuronal activity in rat visual cortex *in vitro*. The second occurrence shows a temporally compressed pattern with increasingly synchronized spikes. Reproduced from Gansel and Singer (2008). **(C)** Gradual temporal compression of repeating spike patterns recorded from multineuronal activity in rat visual cortex *in vitro*, comprising 90,751 individual patterns. The plot shows median ± median deviation of pattern duration depending on pattern repeat and linear regression with coefficient of determination *R*^2^ = 0.98, indicating robust compression. Duration normalized with respect to mean duration per pattern. Reproduced from Gansel and Singer (2008). **(D)** Distribution of durations of repeating spike patterns recorded from multineuronal activity in rat visual cortex *in vitro*. Patterns were collected using a sliding window of 50 ms and statistically validated. Gray line indicates chance level obtained from randomized data. Pattern durations are evenly distributed over the entire time window used for the pattern search, with the exception of a prominent peak below 5 ms, indicating precisely synchronous firing. Taken from Gansel (2014). Panels **(C)** and **(D)** contain data recorded from 10 brain slices.

The hypothesis builds on previous theoretical studies and extends them: while the work of Guyonneau et al. (2005) and Masquelier et al. (2008); Masquelier et al. (2009) demonstrated that single neurons equipped with spike-timing-dependent plasticity can learn to selectively respond at the onset of repeating input patterns (Figure 3A), the hypothesis applies this finding to populations of neurons and investigates its potential consequences on the directed synchronization of cells. Suri and Sejnowski (2002) had shown before that spike-timing-dependent plasticity may help to sustain the propagation of synchronous volleys of distributed discharges in neuronal networks, but did not explain how synchronous activity comes about in the first place. Complementing similar simulations with inhibitory synaptic inputs, Vilimelis Aceituno et al. (2020) explored the development of postsynaptic discharge sequences during learning, emphasizing the concomitant sparsening of responses and pointing to prediction as a possible function of response latency reduction. In an attempt to link the effects of spike-timing-dependent plasticity with the formation of functional cell assemblies, the hypothesis presented here tries to: (a) integrate current knowledge on the mechanisms of synaptic plasticity with plasticity of firing patterns on the network level, (b) explain the emergence of synchronous, distributed spiking activity through learning, (c) relate emerging synchronous activity patterns to cognitive functions, and (d) embed mechanisms and putative functions of a directed synchronization of discharges into the existing philosophical framework, focussing on the “principle of structural coherence” (Chalmers, 1995).

### First prediction: spatial integration

The hypothesis makes several predictions. First, neurons that coordinate their firing in response to a recurring activity pattern do not need to be physically connected, and also do not need to receive the same input signals. All that matters for the mechanism to work is that they are driven by a coherent pattern of activity, i.e., that their inputs are correlated. The ensuing spike time coordination may therefore extend over any distance, providing a simple solution for the problem of how information can be integrated on different spatial scales in the brain. In the visual and somatosensory system, for example, information about sensory stimuli is known to be concurrently represented and processed in several cortical areas (McClurkin et al., 1991; Nicolelis et al., 1998), and neuronal firing has been found to be significantly coordinated across these areas in both sequential (Truccolo et al., 2010) and synchronous (Reed et al., 2008) activity. Such widespread spatial integration of information can be achieved through coordinated adjustments of synaptic strengths in response to correlated inputs, although the tendency of distributed cells to engage in coherent activity patterns is likely to decrease with increasing distance (Ohiorhenuan et al., 2010). It is important to note that the resulting synchronous groups would be different from classical Hebbian cell assemblies as the participating cells would not need to be directly connected (Hebb, 1949; Gerstein et al., 1989), but are fully characterized by the transient (and non-random) synchrony of their discharges (Freiwald et al., 2001; Harris, 2005). From all the cells that happen to be activated by the same repeating excitation pattern, certain subsets could be selected as synchronous groups through coordinated changes in synaptic strengths and latencies (Edelman, 1978) based on the relative onset of a cell’s input signals associated with that excitation pattern. Their joint activity would then signify the onset of that exact neural (or cognitive) event with shortest possible latency. Neurons being recruited into synchronous groups would most likely derive from already existing functional subnetworks (Yoshimura et al., 2005) within which synaptic connections are relatively frequent and strong. The related reshaping of the cortical network is thought to underlie the consolidation of newly acquired “knowledge” and goes along with the fact that neuronal populations in sensory areas exhibit similar activation patterns both spontaneously and in response to sensory input (MacLean et al., 2005; Jermakowicz et al., 2009; Luczak et al., 2009; Luczak and MacLean, 2012; Miller et al., 2014), suggesting that sensory responses are drawn from a limited “vocabulary” of possible activity patterns given by the intracortical functional synaptic connectivity.

### Second prediction: temporal compression

A second prediction is that, given the right plasticity mechanisms, recurring multineuronal spike sequences should progressively be compressed in time if the involved neurons respond to coherent input. Evidence for a temporal compression of repeating activity patterns has indeed been found both *in vitro* (Ikegaya et al., 2004; Gansel and Singer, 2008; Figures 3B,C) and *in vivo* (Euston et al., 2007) and is thought to reflect functional modifications within the neural circuitry. Assuming that the neurons that participate in the spike sequence receive a succession of correlated input signals, the whole process would stop when the cells learned to respond to the very onset of their respective input pattern. If these input patterns have the same temporal origin (which might be related to a sensory stimulus, a motor command, or some other cognitive event), the cells would henceforth respond in unison. In this way, any recurring spatiotemporal activity pattern could eventually be translated into the synchronous spiking of a certain set of cells (cf. Figure 3D).

### Third prediction: sparsening

This, in turn, would result in a significant sharpening of neuronal representations and an increasingly concise layout of information, which is another prediction of the hypothesis: repetitive activation patterns would be transformed to short-latency volleys of synchronous spikes (Suri and Sejnowski, 2002), whereas new spatiotemporal arrangements of signals would provoke temporally dispersed responses. Further, the synchronization would most likely involve a sparsening of related spike events by confining the responses to smaller time windows and concentrating them to the most reliably driven neurons, optimizing energy efficiency (Lennie, 2003; Vilimelis Aceituno et al., 2020) while enhancing the memory capacity of the network (Meunier et al., 1991). The shaping of the functional circuitry through synaptic plasticity might thus contribute to the establishment of a sparse coding scheme (Laughlin and Sejnowski, 2003; Olshausen and Field, 2004) as it appears to be implemented in several sensory cortical areas (Vinje and Gallant, 2000; Weliky et al., 2003; Hromádka et al., 2008; Jadhav et al., 2009; Poo and Isaacson, 2009).

## Neural Correlates of Cognitive Processes

If the “synchrony through synaptic plasticity” hypothesis is correct, groups of synchronously active cells will spontaneously emerge in response to repeating excitation patterns in the brain and consequently indicate a “known” event, suggesting that these synchronous cell assemblies may function as elements of a neuronal code that serve as a correlate of some discrete cognitive content. In what follows, I will speculate about the possible roles of these multineuronal signals in cognitive processes.

### Plasticity, degeneracy, and the hard problem

When relating neural activity to cognitive functions and phenomena, two things need to be considered. The first concern applies to any physical account of conscious experiences and has been addressed as “the hard problem of consciousness” by Chalmers (Chalmers, 1995). Although it is evident that experience arises from a physical basis, we have no satisfactory explanation of why and how it so arises. We may well specify the mechanisms that are responsible for the performance of certain neural or cognitive functions, but why brain activity gives rise to subjective experiences or “qualia” is entirely unclear. In principle, any neuronal process could be instantiated in the absence of experience, or as Chalmers put it: “Experience may *arise* from the physical, but it is not *entailed* by the physical”. It follows that no account of the physical brain processes will tell us why and how they lead to the emergence of qualia. Even if we succeed in mechanistically explaining the ability of the brain to discriminate, categorize and appropriately react to environmental stimuli, shift attention and to deliberately control behavior, learn and to adapt, and to selectively combine, memorize and recall pieces of information, we are still limited to a phenomenological correlation of observed brain dynamics and subjective conscious experiences (Nevertheless, we may hope to identify some psychophysical principles connecting the properties of neuronal processes to the properties of related experiences, as I will exemplify below).

A second problem concerning the relation between neural activity and cognitive functions is caused by the tendency of the brain to display degeneracy (Edelman and Gally, 2001), that is, the ability of structurally different elements to perform the same function or yield the same output. Degeneracy can be found on virtually any organizational level in the brain and is an inherent feature of intra- and intercellular signaling, synaptic plasticity, motor commands, and body movements, and also inter-subject communication (there might be large or sometimes even infinite numbers of ways to transmit the same message, a situation most obvious in language). For instance, different combinations of ionic conductances affecting the integration of dendritic input signals may lead to the generation of identical output signals of a cell (Achard and De Schutter, 2006). On the network level, different configurations of connection strengths and cellular properties may produce the same population activity patterns (Prinz et al., 2004), and different ensembles of neurons may be dynamically configured to initiate the same behavior (Meyrand et al., 1991). Accordingly, we do not lose the perception of a seen object just because its image is slid across the retina—we may continuously perceive it as the same object although changing populations of neurons receive and carry the associated information [eye movements actually serve to *maintain* a stable visual perception (Martinez-Conde et al., 2004)]. Further evidence supports the idea that there is no simple one-to-one relationship between particular activity patterns in the brain and certain cognitive processes: In the visual cortex, neuronal responses to repeated presentations of the same stimulus are highly variable and are strongly determined by the ongoing activity (Arieli et al., 1996; Fiser et al., 2004). In addition, synaptic connections continuingly undergo extensive remodeling (Feldman, 2009; Minerbi et al., 2009), providing the brain with an adaptive yet inherently unstable functional structure. This implies that neural codes may change with time through learning, and that the same activity pattern may be interpreted differently (or evoke a different behavior) later in the day (Eggermont, 1998).

### Synchrony and the principle of structural coherence

Despite these difficulties in establishing well-defined relationships between mind and brain activity, neuronal signaling in the brain is highly organized and far from being random, as are cognitive processes, and some relationships—i.e., some code relating a given activity pattern to a particular cognitive function—must exist between the two. So, given the synchronous activity of a selective set of cells, what could be its cognitive correlate? Chalmers argued that information, when being processed by the brain, has two basic aspects, a physical aspect and a phenomenal aspect, and that its physical representation should have a structure that corresponds directly to the differences between phenomenal states (Chalmers, 1995). If the argument holds, this principle of structural coherence might provide a fundamental link between the characteristics of cognitive processes on one hand and the organization of neuronal activity on the other. Applied to neuronal synchrony, it means that the directed coactivation of a group of distributed cells could be expected to be paralleled by a meaningful convergence of information on the cognitive level, such as reaching a decision or creating a coherent perception based on distributed signals (Singer and Gray, 1995; Engel et al., 1997; Gray, 1999; Singer, 1999a). It has also been suggested that the same mechanisms that mediate the synchronization of distributed discharges are responsible for giving rise to conscious experiences (Engel and Singer, 2001; Singer, 2007). Becoming aware of a percept or an idea necessarily involves a transition from one functional state to a different state, as has been noted by Dennett (Dennett, 2009): “It seems obvious that there has to be a time before which we are not conscious of some item and after which we are conscious of it. In some sense, then, we become conscious of various features of our experience, so there must be some kind of transition, if not arrival at a place or crossing of a boundary, then a change of functional state of one sort or another”. Although it is difficult to demonstrate that conscious perception requires neuronal synchrony, it should be clear that the transient synchronization of a non-random assembly of cells is precisely that sort of neuronal activity that indicates non-random changes in functional states on timescales fast enough to comply with all kinds of cognitive processes, and thus satisfies the principle of structural coherence also with respect to the processes underlying conscious experiences.

### Integration of synchronizing mechanisms

Any functional interpretation of neuronal synchrony has to include and obviously depends on the specific mechanism that is responsible for the synchronization of neuronal activity. One reason for synchronous firing could be concurrent activation through sensory stimuli. In the mammalian visual system, retinal ganglion cells may fire synchronous action potentials simply because they are driven by a common stimulus. In the lateral geniculate nucleus, corticothalamic efferents come into play, and the synchrony of afferent signals becomes a little less trivial (Sillito et al., 1994; Sillito and Jones, 2002). After reaching cortical areas, sensory signals are modulated by the ongoing cortical activity and follow a multitude of diverging and converging pathways within both local and widespread cortical circuits (Figure 2A). In other words, the more central the signals, the more their timing depends on the functional architecture of the network, and the less trivial is the interpretation of neuronal synchrony relating to the sensory stream (Lima et al., 2010).

In the neocortex, neural connections have several inbuilt features that directly support the synchronization of neuronal signals. The most significant feature is the coupling of inhibitory interneurons by gap junctions, forming large, continuous, cell type-specific syncytia (Gibson et al., 1999; Amitai et al., 2002; Fukuda et al., 2006; Figure 2D). Although the amplitude of electrotonic signals quickly falls off with distance, this electrical coupling facilitates the widespread synchronization of rhythmic inhibitory activity, which in turn may constrain the firing of entire populations of pyramidal cells to narrow time windows (Buzsáki and Chrobak, 1995; Beierlein et al., 2000; Whittington et al., 2000; Galarreta and Hestrin, 2001; Bennett and Zukin, 2004). As will be explained below, this mechanism is important for the instantaneous coordination of multineuronal spiking (and hence cognitive processes) on very short timescales. Another feature of many thalamocortical and corticocortical connections is the compensation for differing lengths of a cell’s axonal branches by adjusting the degree of myelination and the diameter of the fibers such that all postsynaptic targets receive the signal at the same time (Innocenti et al., 1994; Salami et al., 2003; Kimura and Itami, 2009; Figure 2B). This makes sense if one assumes that synchrony is a tag for “belonging together” or “being one”. Finally, the cortical network might also be endowed with synchronizing mechanisms other than common input (Figures 2A,B) or synchronous network oscillations (Figure 2E). The “synchrony through synaptic plasticity” hypothesis explains how synchronous cell assemblies could emerge through correlated changes in synaptic efficacies in response to repeating excitation patterns (Figure 2F). The gradual build-up of synchronous groups of cells through learning would be vital for creating “sense out of fact” (Johannesma et al., 1986) and might help to distinguish between familiar and unfamiliar experiences (Suri and Sejnowski, 2002; Korndörfer et al., 2017).

### Selectivity, flexibility, and the learning time barrier

There is, however, a problem: adaptive changes of synaptic connections occur on a much longer timescale than most cognitive processes and are unable to represent changes in sensory information in real time. This inability to instantly reconfigure the functional network in response to afferent signals has been referred to as the “learning-time barrier” (von der Malsburg, 1995) and calls for an additional mechanism that can coordinate multineuronal activity on the timescales on which cognitive processes take place, i.e., within milliseconds. It is obvious that such a fast mechanism can only be realized through dynamic activity patterns emerging from and interacting with the functional neural circuitry. Although any complex activation pattern could in principle serve to selectively excite a certain set of cells while inhibiting others, the most prominent and ubiquitous population pattern that is known to flexibly and coherently modulate the excitability of distributed cells on a milliseconds timescale is synchronous network oscillations. As explained in previous sections, network oscillations naturally arise from the resonant properties of individual neurons and from the interplay of recurrent excitatory and inhibitory connections. The propensity to produce synchronous oscillations is higher when recurrent feedback is strong (Wang, 2010), which adds to the reason why cortical gamma-band oscillations tightly correlate with hemodynamic signals indicating an increase in energy consumption (Niessing et al., 2005). This means that network oscillations are not inherently an energetically “cheap” way to achieve neuronal synchrony, as has been suggested (Buzsáki, 2006). In fact, the “cheapest” way to synchronize the activity of neuronal ensembles would be to arrange functional synaptic connections such that some selected sets of cells will be synchronously activated by a certain preceding (and possibly sparse) activity pattern, as proposed by the “synchrony through synaptic plasticity” hypothesis. Nevertheless do cortical networks readily engage in oscillatory activity, thus serving the need for a fast and dynamic coordination of multineuronal firing. On a mechanistic level, the principal function of synchronous oscillations comes down to a temporal modulation of the effective neuronal connectivity through rhythmic fluctuations of the excitability of cells. Synchronized rhythmic activity and functional synaptic connections thus combine in a complementary way to allow for a spatially and temporally selective transmission of signals and hence for a selective activation of neuronal ensembles at any point in time.

On a more cognitive level, synchronous oscillations effectively reduce the system’s degrees of freedom and restrict the space of possible activity patterns, so as to concentrate on some signals and the information they carry while disregarding others. Indeed do neurons in macaque area V4 that are activated by an attended stimulus engage in enhanced gamma-band synchronization compared with neurons activated by a distracter, pointing to a functional role of synchronous network oscillations in attentional stimulus selection (Fries et al., 2001, 2008). For network rhythms to synchronize the activity of neuron groups, though, it is unimportant if they exhibit a stable phase and frequency—all that matters for an effective coordination of multineuronal signals is the limitation of neuronal discharges to narrow time windows by alternating volleys of synchronous excitation and inhibition (Atallah and Scanziani, 2009; Isaacson and Scanziani, 2011; Nikolic et al., 2013). Could such rhythmic network activity in principle be sufficient for a selective synchronization of neuronal discharges, irrespective of the functional synaptic connectivity? It clearly can not, first because synchronous oscillatory activity is coherent across cell populations (Engel et al., 1990; Jia et al., 2011) and thus lacks the spatial selectivity needed for efficient neural coding, and second because meaningful neuronal synchrony can only arise through experience and learning, which involves adjustments of synaptic efficacies and connections. It is thus evident that while dynamic activation patterns are needed to flexibly arrange synchronous cell assemblies on short timescales, functional adaptations of selected synaptic connections are required to allow for a selective synchronization of cells in the first place. According to the “synchrony through synaptic plasticity” hypothesis, the directed assembly of cells into synchronous groups could be based on the detection of repeating activity patterns and hence on recognizing recurrence as a fundamental property of behaviorally relevant events.

### Directed vs. accidental synchrony

The emergence of selective neuronal synchrony as a potential carrier of information bears the question of how this synchrony is interpreted in subsequent processing stages. It has been argued that parallel synaptic inputs arriving synchronously at a postsynaptic neuron summate more effectively and for this reason transmit their signals more reliably than temporally dispersed inputs (König et al., 1996; Wang et al., 2010). Although pyramidal neurons are indeed more sensitive to coincident inputs especially at high activity levels (Azouz and Gray, 2000, 2003; Prescott et al., 2006), it should be clear that any synchronous discharge pattern in the brain will fan out in both time and space through a multitude of converging and diverging connections, meaning that its impact on downstream cell populations is not determined by the synchronicity of the signals *per se*. The question thus becomes what distinguishes meaningful synchronous discharge patterns from accidental neuronal synchrony, and the only possible answer is their usefulness in interpreting sensory information and generating appropriate behavior (Buzsáki, 2010) by informing downstream populations of neurons about their current functional coherence in an “understandable” and meaningful way. The ability to do so can be expected to depend on prior adaptations of the functional network to enable the immediate recognition and classification of the associated information.

## Discussion

### Neuronal cooperation and the timescale of cell assembly activation

The above arguments on the coordination of neuronal activity through synaptic plasticity and the dynamic gating of signals suggest that the emergence of synchronous cell assemblies and their selective activation may be central to cortical information processing and coding. The following considerations are concerned with the likely organization of these assemblies in time and their composite receptive fields.

### First issue: temporal precision of spike time coordination

The first issue relates to the temporal precision with which neurons could be expected to coordinate their firing. In the past, spike timing was assessed relative to the timing of external stimuli or other spikes emitted by the same cell, a praxis that stems from the classic approach to explain brain function by characterizing the response properties of single cells and simply ignores the coordination of spike events across populations of cells. These studies led to the notion that neuronal spiking was generally unreliable and imprecise, which in turn led to the conclusion that information cannot be represented by the precise timing of spikes. Yet, this view might just be a misconception of the temporal organization of neuronal firing that follows from not taking into account spike timing across cells, which would require the parallel recording of multiple single cells and appropriate measures of their spike time coordination (Masquelier, 2013). We know from combined voltage-sensitive dye imaging and intracellular recordings that the firing of a cortical neuron strongly depends on the present activity pattern in the surrounding area (Tsodyks et al., 1999) and that the large variability of responses to sensory stimulation arises from a quite deterministic interaction of afferent signals with the ongoing activity (Arieli et al., 1996). Sensory evoked neural activity thus represents the modulation of ongoing circuit dynamics by sensory afferents, rather than directly reflecting the structure of the input signal (Fiser et al., 2004). This means that it might be more instructive to relate a neuron’s firing to the activity of its peers than to some external event (Panzeri and Diamond, 2010; Eldawlatly and Oweiss, 2011). The idea that neurons are selectively bound into functional cell assemblies whose activation represents cognitively meaningful units of information and that these assemblies are distinguished by synchronous firing within typically a few milliseconds (cf. Figure 3D) fits with the finding that animals can exploit differences in the timing of cortical signals that are as short as 3 ms to guide decisions (Yang et al., 2008; Hromádka and Zador, 2009).

### Second issue: variability of single cell receptive fields

The second issue concerns the role of single neurons in information representation as part of functional cell assemblies. How independent is the message a single cortical neuron conveys by sending an action potential down its axon from the signaling of others? If it was perfectly related to the firing of any other cell, their signaling would be totally redundant, reducing both the network’s coding capacity and efficiency. If, on the other hand, it was fully independent, then this would imply that it had a fixed meaning that is unmodifiable by collateral signals, like in a labeled line code. However, several arguments suggest that the information carried by the spiking of a particular neuron may not be invariant but be dependent on the functional state of the network as a whole.

First of all, neural connections and synaptic strengths are plastic and subject to continuing modifications (Minerbi et al., 2009), resulting in an ever-changing functional structure of the neuronal network. Together with other factors that control the integration of synaptic inputs on short timescales like ongoing network oscillations and dynamic excitability changes (Civillico and Contreras, 2012), this leads to substantial variability in the receptive fields of neurons (Weinberger, 1995; Wörgötter et al., 1998; Froemke et al., 2007; Sun and Dan, 2009) and thus in the information that could be conveyed by a particular cell. Moreover, neurons in sensory cortical areas may adapt their receptive fields to the properties of the current sensory input (Yeh et al., 2009; Fournier et al., 2011) and were shown to be sensitive also to the larger stimulus pattern (Gallant et al., 1998; Haider et al., 2010), variations of central states (Metherate et al., 1992; Wang and McCormick, 1993), stimuli presented simultaneously in other modalities (Lalanne and Lorenceau, 2004; Driver and Noesselt, 2008; Varga and Wesson, 2013), shifts in spatial and feature selective attention (Womelsdorf et al., 2006; Fritz et al., 2007), and reward values (Shuler and Bear, 2006; Brosch et al., 2011). The responsiveness of single neurons thus depends on the concurrent sensory or cognitive context (Gilbert and Li, 2013). Again, this means that neurons dynamically coordinate their firing and represent information by their joint activity. The synergistic encoding of information by ensembles of neurons, as opposed to single-cell coding, would be favorable also from a theoretical point of view: the variable binding of distributed cells into functionally coherent groups would maximize the repertoire of functional states and in this way dramatically improve the coding capacity of the network. Furthermore, dynamic changes in neuronal firing correlations observed during sensory processing or working memory operations (Vaadia et al., 1995; Sakurai and Takahashi, 2006; Fujisawa et al., 2008; Ohiorhenuan et al., 2010) seem to confirm that individual cells flexibly take part in multineuronal representations. Given the large number of diverging and converging connections in the brain, correlated activity indeed appears to be the rule rather than an exception, as it is commonly caused by shared input (Lampl et al., 1999; Smith and Kohn, 2008; Figure 2B). Yet at the same time, neighboring neurons in sensory cortical areas may actively decorrelate their firing (Gawne and Richmond, 1993; Reich et al., 2001; Smith and Kohn, 2008; Ecker et al., 2010; Renart et al., 2010) in a stimulus-dependent way (Gawne et al., 1996; Vinje and Gallant, 2000; Yen et al., 2007). Cortical neurons thus dynamically change the partners with which they share coherent information, implying that the information that is transmitted by a single cell may vary as a function of the activity of its peers and cannot possibly be decoded without taking into account the concurrent population pattern.

### Binding by synchrony revisited

The dynamic interdependence of neuronal responses and the ensuing formation of multineuronal representations bring us back to the binding problem (Singer and Gray, 1995; von der Malsburg, 1995; Treisman, 1996; Roelfsema, 2006): if a neuron’s firing, taken on its own, does not unambiguously indicate some specific feature or state of the inner or outer world, what are the mechanisms that are responsible for the emergence of defined, meaningful firing patterns across multiple cells, and what is the resulting spatiotemporal structure of these patterns (Singer et al., 1997)? In the preceding sections, I tried to argue that directed synchronous firing within a few milliseconds is what defines the members of a functional cell assembly (Figure 3D), and that their collective activation is what probably defines irreducible units of information. A possible mechanism that could mediate the selective formation of synchronous cell assemblies as carriers of coherent information is given by the “synchrony through synaptic plasticity” hypothesis which consistently explains the signaling of functional relations between neurons by synchronous spikes *and* the computation of these signals: Whenever multiple cells repeatedly receive correlated input, the associated changes of synaptic efficacies may eventually create a set of cells that synchronously respond to a specific activity pattern (Figure 2F) and thus share a common, complex receptive field which cannot be reduced to the receptive fields of single neurons (Johannesma et al., 1986; Brette, 2012).

### Experimental approaches to multineuronal coding

Although much has been said and done since the days of Hebb and the introduction of the cell assembly concept, it appears that we still lack a complete, comprehensive understanding of the dynamic organization of multineuronal activity. The temporal precision of firing and the timescales on which neuronal activity is coordinated are a matter of ongoing debate (deCharms and Zador, 2000; Harris, 2005; Averbeck et al., 2006; Tiesinga et al., 2008). Without a clear characterization of the spatiotemporal structure of concerted neuronal firing on short timescales—that is, the definition of a differentiated signature of neural assemblies—also no superordinate structure possibly representing cognitive processes on longer timescales can be found.

### First requirement: analysis of higher-order firing correlations

What is hence needed is an approach to assess and precisely characterize higher-order correlations among multiple neurons on a moment-by-moment basis. Several methods for the detection and statistical evaluation of recurring multineuronal discharge sequences in parallel recordings have been proposed that provide a precise description of their spatiotemporal organization and allow for a continuous correlation of the activity patterns with the ongoing information processing (Gansel and Singer, 2012; Quaglio et al., 2017; Stella et al., 2019). They are readily applicable and are able to answer the question if neurons fire independently or depending on each other, if repeating spatiotemporal patterns show significant structure, and what the relevant timescales are.

### Second requirement: disentanglement of synchronizing mechanisms

For understanding the neural code, as important as the signature of neural assemblies—the potential information-carrying elements—are the mechanisms that mediate their formation and conversion. It has been argued that network oscillations tend to synchronize action potentials in coherently oscillating cells and so create a signature of functional relatedness (Singer, 1999a; Figure 2E). As already mentioned, they naturally arise from the interplay of recurrent excitatory and inhibitory connections and the resonant properties of individual neurons (Llinás, 1988; Gray and McCormick, 1996; Hutcheon and Yarom, 2000; Cardin et al., 2005). Synchronization of signals is supported locally by the coupling of cells *via* gap junctions (Bennett and Zukin, 2004; Figure 2D). Remote populations may engage in zero phase lag oscillations despite long conduction delays if coupled reciprocally to a relay population of cells (Chawla et al., 2001; Vicente et al., 2008; Viriyopase et al., 2012; Figure 2C), and it has been suggested that thalamic nuclei may play an according role in mediating synchrony among distant brain regions (Jones, 2002; Shipp, 2003; Vicente et al., 2008). Another mechanism by which neuronal activity is organized is the shaping of the functional network through synaptic plasticity. The “synchrony through synaptic plasticity” hypothesis explains the emergence of synchronously active cell ensembles through experience (Figure 2F). How can the influences of these different mechanisms on the generation of precisely timed multineuronal discharge sequences be disentangled and quantified?

As Buzsáki (2006) accurately pointed out, “the acid test for providing a definite proof for the essential role of brain rhythms in computation and brain function would be to selectively eliminate them and examine what is left after the complete lack of oscillatory timing”. However, oscillations are an emergent network property and do not have “receptors” that can be targeted by drugs or other means; only individual neurons do. It is therefore impossible to selectively eliminate a rhythm without fundamentally interfering with the elementary properties of the parts that gave rise to it. Modifying the function of certain receptors for neurotransmitters is likely to radically change the flow of electrical signals in the network and also affect all other activity patterns (Buzsáki, 2006). This criticism applies to the aforementioned experiments that used pharmacological interference and GABAA receptor β3 subunit knock-out mice to disrupt or alter network oscillations, and it also applies to more direct manipulations of the activity of subpopulations of neurons by optogenetic methods. An alternative way to study the organization of neural activity in the absence of neural rhythms could be to record from brain slices: by disconnecting some part of the network from the rest of the brain, chances are high that the remaining network is too small to generate synchronous oscillations (Gansel, 2014). On the downside, neurons encounter a lack of neuromodulators, but these can in principle be applied externally; the important difference between an *in vitro* approach compared to the elimination of oscillatory activity *in vivo* is that receptor function and neuronal excitability can be left untouched and unaffected. Furthermore, recording from an isolated piece of neural tissue allows the investigation of its inherent properties independently of long-range connections and sensory input. This *in vitro* approach closes the gap between *in vivo* recordings and neuronal network simulations and is best suited to validate network models on the basis of real empirical data.

#### Third requirement: multielectrode recordings

The aim to observe coordinated discharges at sub-millisecond time resolution makes it obligatory to simultaneously record from multiple single neurons with multiple electrodes (Buzsáki, 2004; Miller and Wilson, 2008). Until now, multi-site recordings of single-unit spike activity in acute brain slices have been reported on only a few occasions and did not follow any standardized approach. Problems arise in particular when using flat electrodes because spikes can be recorded only from the surface of the slice where most cells are damaged as a result of the slicing procedure, and because spike recording requires auxiliary techniques to assure proper contact of the tissue with the electrodes (Egert et al., 2002). To enable the recording of spikes from cells in the middle of the slice, arrays of long enough, sharpened electrodes would have to be employed (Gansel and Singer, 2013). Such a setup would allow for the observation of a large, random set of neurons of which a subset might participate in a given neuronal assembly. Subsequent analysis, then, would allow for interference of assembly properties. To characterize the organization of local spiking activity in a comprehensive way, it would be necessary to systematically test for coordination of spike timing on several timescales. The discovery of recurring patterns of distributed spiking activity that become progressively compressed in time (Gansel and Singer, 2008; Figures 3B,C) and show precise synchronous firing within typically a few milliseconds while network oscillations and sensory input are absent (Gansel, 2014; Figure 3D) provide direct evidence and strong support for the “synchrony through synaptic plasticity” hypothesis.

## Data Availability Statement

The original contributions presented in the study are included in the article, further inquiries can be directed to the corresponding author.

## Author Contributions

KG developed the research hypothesis and wrote the manuscript.
